# Supplementary material for: Rheological Properties and Sensory Profile of Yoghurt Produced with Novel Combination of Probiotic Cultures
Source: Foods. 2024 Sep 24;13(19):3021. doi: 10.3390/foods13193021 (PMC11476042; doi:10.3390/foods13193021)
Supplement: Supplementary file 1 [file foods-13-03021-s001.zip › foods-3181144-supplementary.pdf]

Supplementary Table S1. Total colour difference (ΔE) calculated between yoghurt samples

ΔE	P1	P2	P3	P4	P5
P1	-	-	-	-	-
P2	0.64	-	-	-	-
P3	0.66	0.14	-	-	-
P4	0.92	0.76	0.87	-	-
P5	0.39	0.53	0.62	0.56	-
